# Endoscopic Endonasal Repair and Reconstruction of Traumatic Anterior Skull Base Defects

**DOI:** 10.1155/2023/6996215

**Published:** 2023-10-06

**Authors:** Isabelle J. M. Williams, Annakan V. Navaratnam, Mark Wilson, Mark S. Ferguson

**Affiliations:** Department of Otolaryngology, Imperial College Healthcare NHS Trust, London, UK

## Abstract

Eighty percent of cerebrospinal fluid leaks (CSF) occur following trauma and complicate 12 to 13% percent of all basilar skull fractures (Prosser, Vender, and Solares, 2011). An endoscopic endonasal approach (EEA) is often the preferred method of repair with greater than 90% success rates (Prosser, Vender, and Solares, 2011). We report a case of a 37-year-old man who presented to our regional level 1 trauma centre with multiple facial injuries. Initial cross-sectional imaging revealed multiple, continuous anterior skull base fractures with associated pneumocephalus. Though initially managed conservatively, the patient represented five days later with unilateral left-sided rhinorrhoea. An endoscopic endonasal repair with a multilayer fat, tensor fascia lata, free mucosal graft, and vascularised local flap reconstruction was undertaken. This case highlights the importance of maintaining a high level of suspicion for delayed CSF leak in traumatic base of skull injury. The EEA enables meticulous dissection and thorough inspection of the skull base, facilitating multilayered repair and reconstruction of defects.

## 1. Introduction

The most common presentation of cerebrospinal fluid (CSF) leak is unilateral, clear rhinorrhoea [1] resulting from a breakdown in the integrity of the dura mater, bony skull base, and sinonasal mucosa [1, 2].

The firm adherence of the dura along the anterior skull base is thought to predispose this region to injury [3]. The anterior skull base lies intimately close to the paranasal sinuses and thus CSF rhinorrhoea is common secondary to sphenoid and ethmoid defects [4]. This provides a potential route for the spread of infection from nose to brain [5].

Skull base fractures remain challenging to treat given their relatively inaccessible location and proximity to important anatomical structures. They are most frequently encountered in polytrauma patients accompanying other intra- and extracranial injuries [6]. The management of CSF leaks in this context requires an interdisciplinary approach involving neurosurgery, otolaryngology, ophthalmology, and plastic surgery [7]. The role of the ear, nose, and throat (ENT) surgeon is paramount when planning a minimally invasive endoscopic approach for the repair of such defects.

We present a case of a 37-year-old patient who presented with traumatic anterior skull base fractures and had a delayed presentation of CSF rhinorrhoea.

## 2. Case Report

A 37-year-old man was admitted to our level 1 regional trauma centre with multiple injuries and an unclear history. On conducting primary survey, he was haemodynamically stable and Glasgow Coma Scale (GCS) was 15. He had obvious left-sided facial injuries and a 10-centimetre left anterior thigh penetrating injury with visible fascial breach.

**Figure 1:**
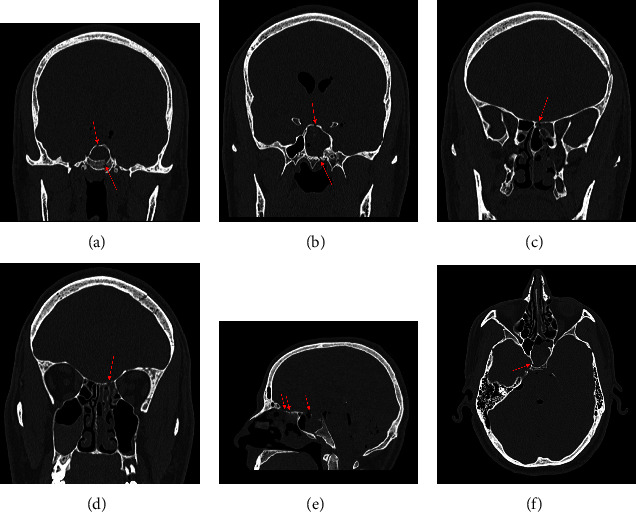
CT images of skull base defects. Red arrows point to skull base defects. (a) Preoperative coronal CT demonstrating defects in left sphenoid sinus and pneumocephalus. (b) Preoperative coronal CT demonstrating defects in left sphenoid sinus. (c) Preoperative coronal CT demonstrating defects in posterior left ethmoid roof. (d) Preoperative coronal CT demonstrating defects in posterior left anterior ethmoid roof. (e) Preoperative sagittal CT demonstrating multiple defects in the roof of the left ethmoid and sphenoid sinuses. (f) Axial CT demonstrating fracture of posterior wall of sphenoid sinus.

A computed tomography (CT) head scan revealed multiple calvaria and skull base fractures with resultant pneumocephalus, subdural, and subarachnoid haemorrhages with signs of raised intracranial pressure (ICP) (Figures 1(a)–1(f)).

On the second day of admission, the patient underwent operative management of his thigh injury. His head injuries were treated conservatively with bedrest, appropriate analgesia, and laxatives. The patient was discharged on day six with appropriate safety netting advice.

The patient represented five days later with left-sided, clear rhinorrhoea and a low-pressure headache. On assessment, he had no focal neurology or localising signs to suggest impending herniation. Repeat CT head imaging revealed worsening pneumocephalus. Nasal discharge was sent for formal CSF analysis and tested positive for beta-2 transferrin. A multidisciplinary team discussion between neurosurgery and ENT concluded that the skull base fractures in the left sphenoid sinus and ethmoid roof were amenable to an endoscopic endonasal approach (EEA) using image-guided navigation.

### 2.1. Operation

**Figure 2:**
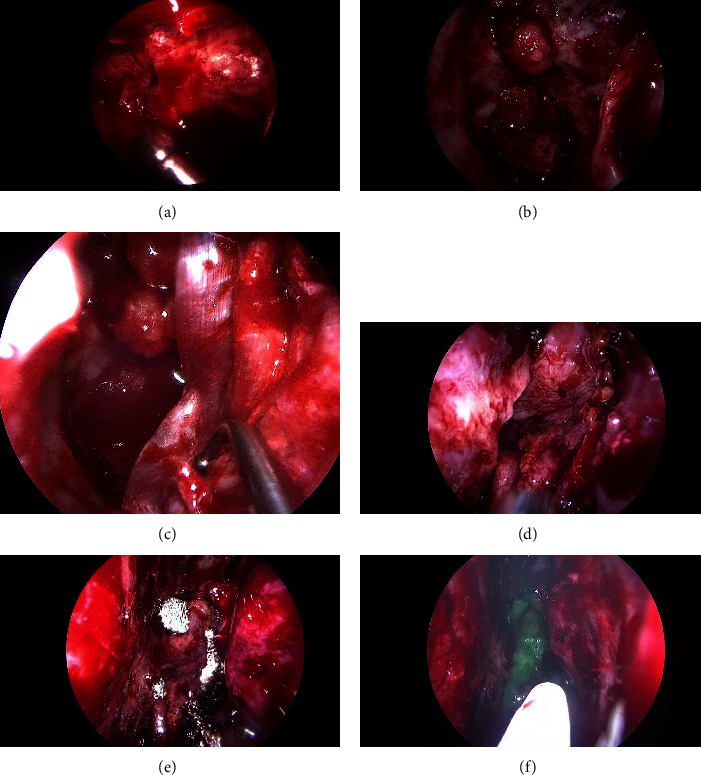
Intraoperative repair and reconstruction of defects: *Tensor fascia lata (TFL)* and *nasoseptal flap (NSF)*. (a) Fracture in posterior wall of left sphenoid sinus. (b) Fat in posterior sphenoid sinus wall defect. (c) TFL graft. (d) NSF placed onto TFL graft. (e) Surgicel being placed onto edges of NSF. (f) Dural sealant being sprayed onto NSF.

The left anterior skull base was explored after resection of the left middle turbinate, a left uncinectomy, left ethmoidectomy, and a wide left sphenoidotomy. A continuous skull base fracture with active CSF leak (Figure 2(a)) was found extending from the posterior wall of the left sphenoid sinus to the ethmoid roof and left lateral lamella of the cribriform plate, with transection of both the left anterior and posterior ethmoid arteries and exposure of the cavernous sinus in the posterior wall of the sphenoid sinus. Control of the cavernous sinus bleeding was achieved with the application of SURGIFLO® haemostatic matrix and bipolar diathermy.

A posteriorly based left nasoseptal flap was raised and placed into the nasopharynx (Figure 2(d)). The left middle turbinate was used to harvest free mucosal grafts. Tensor fascia lata (TFL) and fat were harvested from the right leg via a lateral incision. Left-sided sphenoid and posterior ethmoid defects were closed with 3-layer closure: Fat, TFL, nasoseptal flap, and hydrogel dural sealant (Adherus Autospray ET) (Figures 2(b)–2(f)). The mid-anterior ethmoid and lateral lamella defects were closed again with a 3-layer closure: Fat, TFL, free mucosal graft (from middle turbinate), and hydrogel dural sealant.

**Figure 3:**
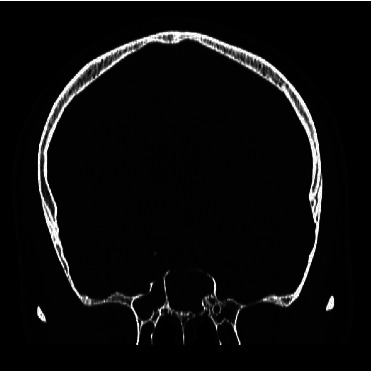
Postoperative coronal CT image demonstrating resolution of pneumocephalus.

An absorbable haemostat dressing (Surgicel® (oxidized regenerated cellulose)) was applied around the edges of the nasoseptal flap and free mucosal graft (Figure 2(e)). The nose was then packed with bioabsorbable nasal dressing (Nasopore Forte, Polyganics, Groningen, the Netherlands)). A lumbar drain (LD) was placed at L4-5, connected to a Becker® External Drainage and Monitoring System. The patient was admitted to the neurosurgical ward for monitoring, bedrest, and nursing at 30 degrees. Postoperative CT imaging showed resolution of pneumocephalus (Figure 3). The LD was removed on day six after the procedure and the patient was discharged on day seven.

### 2.2. Follow-Up and Outcome

Our patient re-presented nine days following initial discharge with worsening headaches and self-reported fevers. He denied nausea, vomiting, neck stiffness, or photophobia. A CT head scan showed no ongoing communication with the paranasal sinuses and resolution of the pneumocephalus. He was admitted for observation and was discharged 24 hours later. On follow-up, two months later, he was free from complications.

## 3. Discussion

Skull base fractures are complex and may be overlooked in the polytrauma setting, whereby other life-threatening injuries may take precedence. CSF leak may be delayed and therefore clinicians must maintain a high index of suspicion in cases of clear rhinorrhoea following head trauma.

### 3.1. Diagnosis

CSF rhinorrhoea occurs due to trauma in 80–90% of cases [8, 9] and most commonly presents as a watery nasal discharge followed by nasal obstruction [10]. Of note, Naidu et al. found that 19% of patients with skull base fractures have no clinical signs or symptoms of CSF leak at presentation [11]. Indeed, a retrospective review by Phang et al. found that only half of traumatic CSF leaks became evident within 48 hours of injury, with 95% of cases manifesting within three months [7].

Diagnosis relies on both clinical and radiological assessment. Evidence of pneumocephalus and a skull base defect or fracture on CT imaging will usually be identified. However, with a sensitivity of 94–100% and specificity of near 100%, the beta-2 transferrin assay has become the gold standard for detection of CSF leakage [12].

### 3.2. Treatment

Various treatment modalities have been utilised for the management of CSF leak. Most early leaks heal with conservative management. In one case series of 735 patients, Bell et al. found that 85% of patients with identified CSF leak experienced uncomplicated, spontaneous resolution of their leak [13]. Such an approach involves bed rest, head elevation, and avoidance of exertional activities that increase ICP including coughing, retching, and Valsalva manoeuvres.

CSF diversion can be achieved with LD, encouraging spontaneous closure through reduction of CSF pressure. Fan et al. reported a success rate of 53% in a case series of 17 patients managed with LD [14]. However, a recent systematic review focusing on the clinical and pathological features of post traumatic meningitis by La Russa et al. concluded that unrepaired CSF leaks may persist over days, increasing the risk of developing meningitis and other neurological sequelae, especially when exceeding one week [5].

Endoscopic closure of CSF leak has revolutionised the surgical management of CSF rhinorrhoea and has reduced the morbidity associated with it [15]. In a large series of 193 patients with CSF rhinorrhoea, Banks et al. report an overall success rate of 98% with endoscopic, endonasal repair of the skull base defects [4]. Moreover, Senior et al. report an overall success rate of 90% and complication rate of 2.5% in their single institutional analysis of 522 cases [16]. More recently, smaller series corroborate these findings with reported success rates of up to 100% [2, 17, 18].

Major advantages of the EEA include low retraction injury to brain cortex and preservation of sense of smell as well as excellent access and visualisation of the sphenoid parasellar and posterior ethmoid regions, allowing for precise graft placement and thus durable reconstruction [19].

### 3.3. Techniques

The anterior skull base defects are well suited to repair via the EEA. A range of options are available to the endoscopic surgeon depending on the size of the skull base defect, volume of leak, and location.

Simple harvested free mucosal grafts (from the turbinates) can be used for small cribriform defects where thin bone prevents multilayered reconstruction [1, 8]. Fat grafts and temporalis fascia grafts are also options for free autografts in skull base reconstruction but require separate incisions for harvest. The posteriorly pedicled nasoseptal flap, based on the posterior septal artery branch of the sphenopalatine artery, is the workhorse of the vascularised intranasal local flap and can cover 50% of the anterior skull base [20].

In a study of 42 patients undergoing endoscopic multilayered repair of traumatic CSF leak, TFL grafts, fat plugs, and nasoseptal flaps were used in 100%, 83.3%, and 69% of cases, respectively [17]. Such approach achieved a success rate of 97%, with only one patient requiring a further second repair [17].

Furthermore, a recent retrospective review of four anterior skull base defects repaired via the EEA demonstrated effective repair and durable reconstruction of complex, multifocal, traumatic anterior skull base defects using a multilayer TFL apposition technique and pedicled nasoseptal flap coverage [18]. Repair was successful in all patients with only one requiring reoperation for tension pneumocephalus [18].

The use of perioperative LD is controversial according to most series. Locatelli et al. performed LD in only 2/135 patients with intracranial hypertension [2]. In contrast, all patients had LD prior to surgery in the small Sheth et al. series [18]. Whilst evidence suggests that perioperative LD in the context of vascularised nasoseptal flap closure significantly reduces the rate of postoperative CSF leak [21], it is unclear if LD on its own can prevent CSF leak following traumatic base of skull injuries.

Where there are large defects, or if total sphenoidotomy is needed to visualise and access the skull base, the nasoseptal flap may inadequately cover the defect. In such cases, flaps can be extended by harvesting mucosal tissue from the nasal cavity floor and under the inferior turbinate [18]. The anterior ethmoid artery septal flap can also be utilised to repair more anteriorly sited skull base defects [22].

In this reported case, a composite multilayered repair was successfully utilised for the posterior and anterior segments of the skull base fracture, with a nasoseptal flap used posteriorly and free mucosal grafts anteriorly, in addition to fat grafts, TFL, and a hydrogel dural sealant.

## 4. Conclusion

This case supports the rationale that endoscopic management of CSF leak can be safe and effective. We illustrate the importance of adapting the approach and type of technique used for the site and size of the defects, creating a multilayer, durable reconstruction.

## 5. Key Points

1. Skull base injuries are significant, coinciding with traumatic brain injury as well as neurological, sensory, and potentially infectious complications
2. CSF leak can complicate base of skull fractures, presenting most frequently as CSF rhinorrhoea
3. Presentation of CSF leaks after the skull base injuries can be delayed and clinicians should maintain a high level of suspicion for late presentations of clear rhinorrhoea following head injuries
4. Controversy remains regarding the timing of CSF leak repair versus a watch-and-wait approach
5. The EEA prevents the morbidity associated with open craniotomy whilst allowing for careful dissection and multilayer closure in most anterior skull base injuries

## Data Availability

No data were used to support this study.
